# Symptomatology during previous SARS-CoV-2 infection and serostatus before vaccination influence the immunogenicity of BNT162b2 COVID-19 mRNA vaccine

**DOI:** 10.3389/fimmu.2022.930252

**Published:** 2022-10-14

**Authors:** Sabryna Nantel, Benoîte Bourdin, Kelsey Adams, Julie Carbonneau, Henintsoa Rabezanahary, Marie-Ève Hamelin, Deirdre McCormack, Patrice Savard, Yves Longtin, Matthew P. Cheng, Gaston De Serres, Jacques Corbeil, Vladimir Gilca, Mariana Baz, Guy Boivin, Caroline Quach, Hélène Decaluwe

**Affiliations:** ^1^ Cytokines and Adaptive Immunity Lab, Sainte-Justine University Hospital and Research Center, Montréal, QC, Canada; ^2^ Microbiology, Infectiology and Immunology Department, Faculty of Medicine, University of Montréal, Montréal, QC, Canada; ^3^ Clinical Department of Laboratory Medicine, Infection Prevention and Control, Sainte-Justine University Hospital and Research Center, Montréal, QC, Canada; ^4^ Infectious Disease Research Center, Université Laval, Québec City, QC, Canada; ^5^ Centre Hospitalier Universitaire de Québec - Université Laval Research Center, Québec City, QC, Canada; ^6^ Microbiology, Infectiology and Immunology Department, Université Laval, Québec City, QC, Canada; ^7^ Immunopathology Department, Montreal University Hospital and Research Center, Montréal, QC, Canada; ^8^ Infectious Diseases Service, Department of Medicine, Jewish General Hospital, Montréal, QC, Canada; ^9^ Biological and Occupational Risk, Divisions of Infectious Diseases and Medical Microbiology, Departments of Medicine and Laboratory Medicine, McGill University Health Center, Montréal, QC, Canada; ^10^ Biological and Occupational Risk, Institut National de Santé Publique du Québec, Québec City, QC, Canada; ^11^ Preventive and Social Medicine Department, Université Laval, Québec City, QC, Canada; ^12^ Molecular Medicine Department, Université Laval, Québec City, QC, Canada; ^13^ Pediatric Immunology and Rheumatology Division, Department of Pediatrics, University of Montréal, Montréal, QC, Canada

**Keywords:** SARS-CoV-2, COVID-19, mRNA vaccination, hybrid immunity, adaptive immune response

## Abstract

Public health vaccination recommendations for COVID-19 primary series and boosters in previously infected individuals differ worldwide. As infection with SARS-CoV-2 is often asymptomatic, it remains to be determined if vaccine immunogenicity is comparable in all previously infected subjects. This study presents detailed immunological evidence to clarify the requirements for one- or two-dose primary vaccination series for naturally primed individuals. The main objective was to evaluate the immune response to COVID-19 mRNA vaccination to establish the most appropriate vaccination regimen to induce robust immune responses in individuals with prior SARS-CoV-2 infection. The main outcome measure was a functional immunity score (zero to three) before and after vaccination, based on anti-RBD IgG levels, serum capacity to neutralize live virus and IFN-γ secretion capacity in response to SARS-CoV-2 peptide pools. One point was attributed for each of these three functional assays with response above the positivity threshold. The immunity score was compared based on subjects’ symptoms at diagnosis and/or serostatus prior to vaccination. None of the naïve participants (n=14) showed a maximal immunity score of three following one dose of vaccine compared to 84% of the previously infected participants (n=55). All recovered individuals who did not have an immunity score of three were seronegative prior to vaccination, and 67% had not reported symptoms resulting from their initial infection. Following one dose of vaccine, their immune responses were comparable to naïve individuals, with significantly weaker responses than individuals who were symptomatic during infection. These results indicate that the absence of symptoms during initial infection and negative serostatus prior to vaccination predict the strength of immune responses to COVID-19 mRNA vaccine. Altogether, these findings highlight the importance of administering the complete two-dose primary regimen and following boosters of mRNA vaccines to individuals who experienced asymptomatic SARS-CoV-2 infection.

## Introduction

As of March 2022, more than 450 million people have been infected by the Severe Acute Respiratory Syndrome Coronavirus 2 (SARS-CoV-2), leading to over six million deaths from Coronavirus Infectious Disease 2019 (COVID-19) (1–3). The range of symptoms associated with SARS-CoV-2 infection is highly diverse, and the scale of symptoms spans from asymptomatic to severe (4). Public health vaccination guidelines of adults who have recovered from SARS-CoV-2 infection differ depending on the country. In Canada and the United States, the National Advisory Committee on Immunization (NACI) and the Centers for Disease Control and Prevention (CDC) recommend a two-dose primary regimen for every adult (5–8), regardless of previous infection status, while many countries such as France, Germany, Italy, and ten other countries from the European Union consider that infection with SARS-CoV-2 is equivalent to one vaccine dose (9). It remains to be established if vaccine requirements for previously infected subjects differ from those for naïve individuals.

Up to one year after their infection, recovered individuals present strong and slowly decaying humoral and cellular responses (10–13). Importantly, it was suggested that the strength of the immune response following natural infection is proportional to the disease severity (14–16). In previously infected individuals, one vaccine dose a few months after infection acts as a booster of immune responses (17, 18). It elicits much stronger B- and T-cell-specific responses in previously infected individuals, compared to naïve individuals, with minimal benefits observed when a second dose is given 21 days after the first one (10, 11, 18–22). However, it is unknown if individuals who were previously infected, but did not experience symptoms, show similar heightened immune responses after a single mRNA vaccine dose. We hypothesized that the symptom severity during natural infection impacts the response to vaccination and that individuals who did not experience symptoms during infection may present suboptimal protection after one dose of vaccine.

In this report, we used three biomarkers of immunity to characterize an effective immune response to SARS-CoV-2. We assessed (1) circulating anti-RBD IgG levels, (2) functional serum capacity to neutralize live SARS-CoV-2 virus and (3) IFN-γ secretion by memory T cells in response to mega pools of SARS-CoV-2 peptides in a cohort of naïve and previously infected individuals before and after COVID-19 mRNA vaccination. We compared the impact of symptoms at the time of primary infection and serology at enrollment (prior to vaccination) on the immune responses to one and two vaccine doses.

## Methods

### Study participants

Our cohort consisted of 55 previously infected health care workers (HCWs) who were recruited following a PCR-confirmed SARS-CoV-2 infection as part of the RECOVER study (n=569) (23), and 14 naïve HCWs with neither history of SARS-CoV-2 infection nor the presence of anti-N or anti-RBD antibodies against SARS-CoV-2 prior to vaccination with BNT162b2 mRNA vaccine (Pfizer-BioNTech, 30 μg per dose). PCR screening was not part of the study itself, and infections were therefore detected through provincial and institutional PCR screening policies, including following exposure to a confirmed SARS-CoV-2 infection case. Participants included in this study were selected based on their reported symptomatology at the time of infection and their serostatus at enrollment in the study. All RECOVER subjects who were asymptomatic (n=28/569) and for whom samples were available before and after vaccination were included (n=19). Participants who were symptomatic during infection (n=26) and for whom samples were available before and after each dose of vaccine were selected from the rest of the RECOVER cohort based on their serostatus at enrollment after being matched with participants from the asymptomatic group for sex, age, ethnicity, time since infection, time between infection and vaccination as well as time between vaccination and each sample available. Characteristics of the study population are shown in (**Table 1 in Supplemental 1**). The severity of the initial SARS-CoV-2 infection was determined using the WHO clinical progression scale (4). Blood samples for humoral and cellular immunity were collected at enrollment and at different time points after vaccination as described in **Figure S1 in Supplemental 1**. Participants were recruited from August 17, 2020, to April 8, 2021, and followed for one year after enrollment.

### Sample collection and processing

Blood samples were collected into serum separation tubes (SST™, BD) and acid–citrate–dextrose tubes (ACD, BD). They were shipped to the Rare Pediatric Disease (RaPID) biobank at the Sainte-Justine University Hospital Research Center, where serum and peripheral blood mononuclear cells (PBMCs) were isolated according to standard operation procedures (SOPs) using SepMate™ tubes (Stemcell Technologies, Canada) for PBMCs isolation. Serum was cryopreserved at -80°C, and PBMCs were cryopreserved in complete RPMI (Gibco) with 10% DMSO and stored in liquid nitrogen until used.

### Exposures and outcome

Exposure was defined as vaccination with Pfizer BioNTech BNT162b2 mRNA vaccine. Vaccine administration was not part of the study itself, as participants received their vaccines through routine public health programs. The primary outcome was the multi-functionality of the immune response developed after each vaccine dose, represented as an immunity score from zero to three. One point was attributed for each of the following immune assays above positive threshold: anti-RBD IgG levels by ELISA, plasma capacity to neutralize live SARS-CoV-2 (ancestral strain) by micro-neutralization assay, and IFN-γ secretion by PBMCs in response to SARS-CoV-2 mega pool of Spike peptides, determined by ELISpot.

### Enzyme-linked immunosorbent assay

An ELISA was performed on serum samples to detect specific IgG antibodies for the Receptor Binding Domain (anti-RBD) of SARS-CoV-2 Spike Glycoprotein as previously described (24, 25). Seropositivity was defined as an OD_490_ ratio greater than one with the experimentally determined cut-off, which corresponds to the mean of 10 negative controls plus three times the standard deviation of the negative controls for each experiment. This anti-RBD IgG ELISA was validated on the Canadian National Microbiology Laboratory SARS-CoV-2 National Serology Panel (23, 26). Absence of previous infection to SARS-CoV-2 was confirmed in the naïve cohort by performing an ELISA detecting specific IgG antibodies for the Nucleoprotein (anti-N) of SARS-CoV-2 and the anti-RBD ELISA before vaccination.

### Micro-neutralization assay

Neutralizing antibody titers were assessed using SARS-CoV-2/Québec City/21697/2020 strain (ancestral Wuhan-1 like SARS-CoV-2), isolated from a clinical sample in March 2020 in Québec City, Canada. Two-fold dilutions of heat-inactivated serum were prepared, starting with 1:20 dilution. Equal volumes of serum and virus were mixed and incubated for one hour at room temperature. The residual infectivity of those mixtures was assessed in quadruplicate wells of African green monkey kidney E6 cell line (Vero ATCC^®^ CRL-1586™). Neutralizing antibody titers were defined as the reciprocal of the serum dilution that completely neutralized the infectivity of 100 TCID_50_ of SARS-CoV-2, determined by the absence of cytopathic effect on cells after four days (27). The neutralizing antibody titer is calculated using the Reed/Muench method (28). These studies were performed in the Containment Level 3 (CL3) laboratory at the Centre Hospitalier Universitaire de Québec - Université Laval Research Center.

### Enzyme-linked immunospot assay

Cell-mediated immune (CMI) response was estimated by ELISpot. Frozen PBMCs were rapidly thawed and rested overnight. They were then stimulated with 1 µg/mL of spike glycoprotein (S), nucleocapsid protein (NCAP) or membrane protein (VME1) mega pools of SARS-CoV-2 peptides from the ancestral Wuhan-1 like strain (JPT Peptide Technologies, JPT) as described elsewhere (29). Spots were revealed using BIO-RAD Alkaline Phosphatase Conjugate Substrate Kit. The resulting ELISpots were analyzed using CTL ImmunoSpot^®^ S5 UV Analyzer (Cellular Technology Ltd, OH). Culture media and phytohemagglutinin (PHA, Sigma) were used as negative and positive controls, respectively. The positive threshold response was defined as more than 25 spot-forming units (SFU) per million cells.

### Activation induced markers assay

Thawed and rested PBMCs were stimulated for 20 hours with 1 µg/mL of the CD4-Spike peptides mega pool developed and synthesized by the Sette laboratory (30, 31). Constituted of 246 HLA-class II restricted 15-mers spanning the whole Spike Glycoprotein, the CD4-Spike mega pool stimulates both CD4 and CD8 T cells. Cells were then stained with panels of fluorescent monoclonal antibodies (**Table 3 in Supplemental 1**). Data were acquired with an LSR FORTESSA II with High Throughput Sampler (HTS) from BD Biosciences (**Figure S2 in Supplemental 1** for gating strategy). FlowJo software, version 10.7.1, was used to perform all data analysis. Fluorescence Minus One (FMO), unstimulated (negative control) and PMA-Ionomycine (positive control) conditions were used to set the gates for each participant. Results are represented by the frequency of activated cells following peptide stimulation minus the frequency of activated cells in the unstimulated condition.

### Statistics

All statistical tests were performed using Prism 9, version 9.2.0 (2021 GraphPad Software, LLC) and data were displayed as median [IQR, 25^th^-75^th^ percentile]. Mann-Whitney or Kruskal-Wallis unpaired nonparametric tests were used to evaluate statistical significance between groups for variables that did not follow a normal distribution. Simple linear regression is presented with Pearson correlation (R^2^) and P-value (p). Significance was set as *P < .05; **P < .01; ***P < .001; ****P < .0001.

### Ethics

RECOVER protocols were approved by the Research Ethics Board (REB) at the Sainte-Justine University Hospital and Research Center under study MP-21-2021-3035 and in each of the five participating centers in the Province of Québec. Written informed consent was obtained from all participants during the recruitment period, and ongoing consent was reviewed at each subsequent visit.

## Results

### Participants characteristics and natural immunity to SARS-CoV-2 infection

The RECOVER cohort consists of 569 HCWs infected with SARS-CoV-2 during the first and second pandemic waves in the province of Québec, Canada (23). Based on surveillance data from public health authorities, most participants enrolled in the study were infected when the original Wuhan-1-like strain was in circulation (23). Some HCWs enrolled were infected at the beginning of 2021, when both the original strain and the Alpha variant were in circulation in our province. Overall, 95% of participants had symptoms at the time of diagnosis (n=541), and very few were asymptomatic (n=28). Among asymptomatic subjects, only 43% were seropositive at enrollment, in contrast to 77% of symptomatic individuals (**Figure S3A in Supplemental 1**). The presence of symptoms at the time of initial infection correlated with more robust anti-RBD IgG levels (median [IQR, 25^th^-75^th^ percentile], 1.5 [1.0-2.2] vs. 0.7 [0.5-1.5], P<.0001) in contrast to subjects who did not experience symptoms. Further, antecedent infection led to long-lasting humoral immune responses in most HCWs (up to 12 months post-infection; **Figure S3B in Supplemental 1**). Disease severity, assessed from 1 to 5 using the WHO clinical progression score (4), increased the strength of this humoral response in HCWs (**Figure S3C in Supplemental 1**). Cell-mediated immune responses to the major components of SARS-CoV-2 were also assessed in almost half of the RECOVER cohort (n=200) by ELISpot, prior to any vaccination. Responses to Spike, NCAP and VME1 were present and maintained up to one year after natural infection in the majority of unvaccinated participants (84%, 80% and 72% respectively), with the most potent response being specific for Spike antigens (85.0 [45.0-177.5] SFU/10^6^ PBMCs, compared to 65.0 [30.0-110.0] and 55.0 [20.0-115.0] SFU/10^6^ PBMCs for NCAP and VME1) (**Figure S4 in Supplemental 1**). From this cohort, we selected a subset of symptomatic and asymptomatic individuals (n=55) to study the impact of previous infection on the immune response to vaccination. Of that subset, 93% (51/55) were infected when only the Wuhan-1-like strain was circulating. All subjects received the BNT162b2 Pfizer BioNTech COVID-19 vaccine, with a first dose administered (mean time ± SD) 8.4 ± 2.3 months (range: 0.8-13.1 months) after infection (**Table 2 in Supplemental 1**). The second dose was offered 16 ± 6 weeks after the first dose. Samples were collected before vaccination, around 45 ± 25 days and 57 ± 30 days after the first and second dose, respectively. They were compared to 14 previously naïve individuals before vaccination, 29 ± 2 days after the first vaccine dose and 35 ± 5 days after the second dose.

### One vaccine dose strongly boosts humoral and cellular responses in recovered individuals

**Figure 1:**
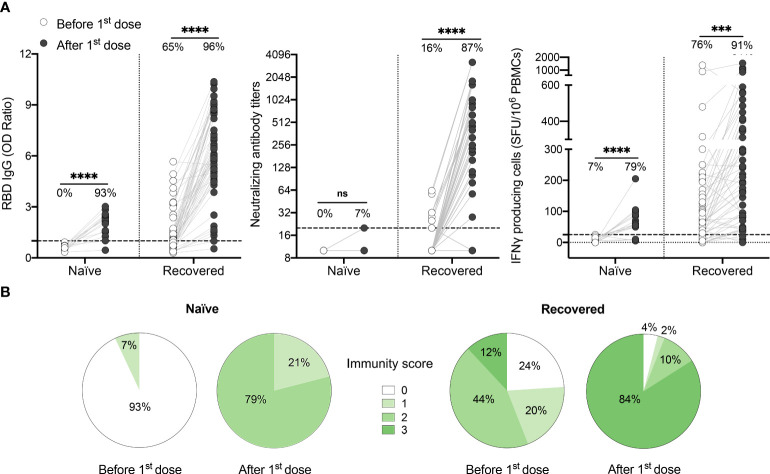
One dose of COVID-19 mRNA-vaccine strongly boosts SARS-CoV-2-specific humoral and cellular responses in most HCWs who recovered from SARS-CoV-2 infection. **(A)** SARS-CoV-2 Spike RBD–specific binding IgG levels assessed by ELISA (left panel), SARS-CoV-2 neutralizing antibody titers (middle panel), IFN-γ secreting cells per million in response to SARS-CoV-2 Spike glycoprotein peptides (right panel) assessed by ELISpot for naïve (n=14) and recovered HCWs (n=55) before (white circles) and after one dose of vaccine (black circles). Dashed black lines indicate the positive threshold value, and the percentage of individuals with responses above positive threshold value is indicated for each time point. Statistical significance was assessed by Mann-Whitney tests. Not significant (ns) P >.05; ***P <.001; ****P <.0001. **(B)** Pie charts of calculated immunity score for naïve and recovered HCWs before and after one dose of vaccine.

Prior to vaccination, most recovered HCWs presented detectable anti-RBD IgG levels (1.1 [0.6-1.7]) and IFN-γ responses to Spike antigens (70.0 [25.0-155.0] SFU/10^6^ PBMCs), while serum neutralization capability was mainly absent or low. Following one vaccine dose, recovered individuals demonstrated a striking increase in their anti-RBD IgG levels, neutralization capacity and cell-mediated immune response (1.1 [0.6-1.7] to 6.1 [4.8-8.7], P<.0001, 10.0 [10.0-10.0] to 254.0 [113.0-640.0] P<.0001, 70.0 [25.0-155.0] to 195.0 [70.0-360.0] SFU/10^6^ PBMCs, P<.001) (**Figure 1A**). In contrast, only one naïve individual showed detectable neutralization titers following the first vaccine dose. In naïve subjects, the breadth of humoral and cellular immune response was also significantly reduced compared to previously infected subjects, with reduced IgG levels (2.2 [1.5-2.5]) and a lower number of IFN-γ secreting cells (60.0 [40.0-92.5] SFU/10^6^ PBMCs) compared to recovered HCWs (6.1 [4.8-8.7] IgG OD ratio and 195.0 [70.0-360.0] SFU/10^6^ PBMCs respectively; P<.0001 and P=.003) (**Figure S5 in Supplemental 1**).

It has been demonstrated that a well-coordinated humoral and cellular immune response, with the development of both antibody neutralizing capacity and cell-mediated immune responses, protects against severe disease (14, 15). In previously infected HCWs vaccinated with one dose, we observed that the strength of the cellular response, as assessed by ELISpot assay, correlates positively with both RBD-binding IgG and SARS-CoV-2 neutralizing antibody titers (**Figure S6 in Supplemental 1**), with the strongest correlation being between IFN-γ response and neutralization capacity. This suggests that vaccine-induced immune protection relies on a synchronized adaptive immune response (32, 33). To better depict the global functionality of the immune response of each subject, we calculated an immunity score based on the presence or absence of RBD-binding antibodies, SARS-CoV-2 neutralizing antibodies and CMI response for each participant. We showed that none of the naïve individuals had a global immunity score of three after one dose of vaccine (**Figure 1B**). In contrast, 84% of the previously infected individuals reached the maximal immunity score, the remainder showing an incomplete response and 6% demonstrating nearly no response (**Figure 1B**).

### Symptomatology at the time of infection impacts the immune response to one vaccine dose

**Figure 2:**
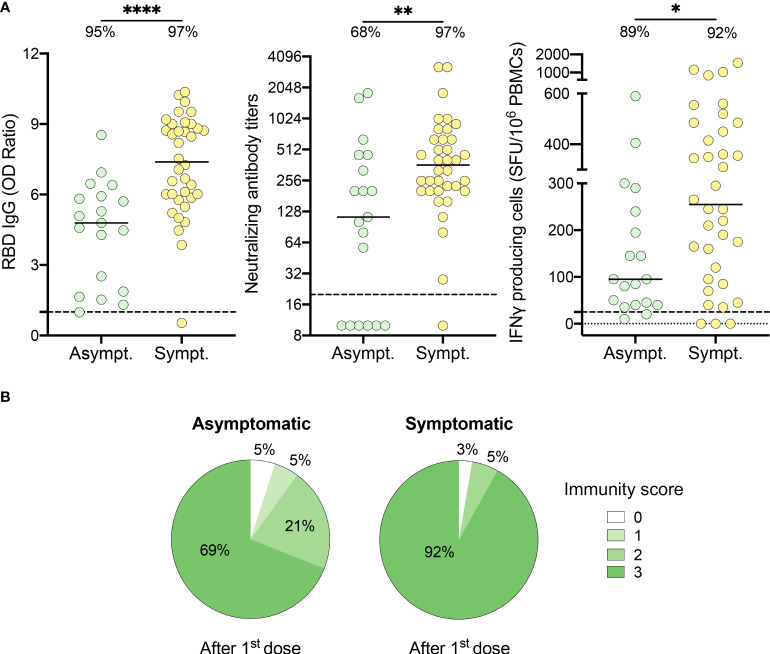
HCWs who recovered from an asymptomatic SARS-CoV-2 infection demonstrate partial immune response after one dose of vaccine. **(A)** SARS-CoV-2 Spike RBD–specific binding IgG levels assessed by ELISA (left panel), SARS-CoV-2 neutralizing antibody titers (middle panel), IFN-γ secreting cells per million in response to SARS-CoV-2 Spike glycoprotein peptides (right panel) assessed by ELISpot for asymptomatic (Asympt.) (n=19) and symptomatic (Sympt.) (n=36) recovered HCWs after one dose of vaccine. Dashed black lines indicate the positive threshold value, and the percentage of HCWs with responses above positive threshold value is indicated for each assay. Horizontal bars indicate the median of each group. Statistical significance was assessed by Mann-Whitney tests. *P <.05; **P <.01; ****P <.0001. **(B)** Pie charts of calculated immunity score for asymptomatic (left panel) and symptomatic (right panel) recovered HCWs after one dose of vaccine.

As 16% of recovered participants had an incomplete immune response after one dose of vaccine, we questioned if the presence of symptoms at the time of infection influenced the immune response to vaccination. Compared to symptomatic subjects, individuals who remained asymptomatic during their initial infection exhibited reduced anti-RBD IgG levels (7.4 [5.9-9.0] vs. 4.8 [1.9-5.9], P<.0001), neutralizing antibody titers (361.5 [202.0-640.0] vs. 113.0 [10.0-453.0], P=.007) and IFN-γ secreting cells (255.0 [101.3-476.3] vs. 95.0 [40.0-240.0] SFU/10^6^ PBMCs, P=.02) (**Figure 2A**). Importantly, asymptomatic individuals were less likely to neutralize SARS-CoV-2 after one dose of vaccine compared to symptomatic subjects (68% vs. 97%, P=.007). Furthermore, 92% of symptomatic individuals showed a multi-functional immune response with an immunity score of three after their first dose of vaccine compared to 69% of asymptomatic individuals (**Figure 2B**).

### Serology status six months after infection influences the strength of immune responses following one vaccine dose

**Figure 3:**
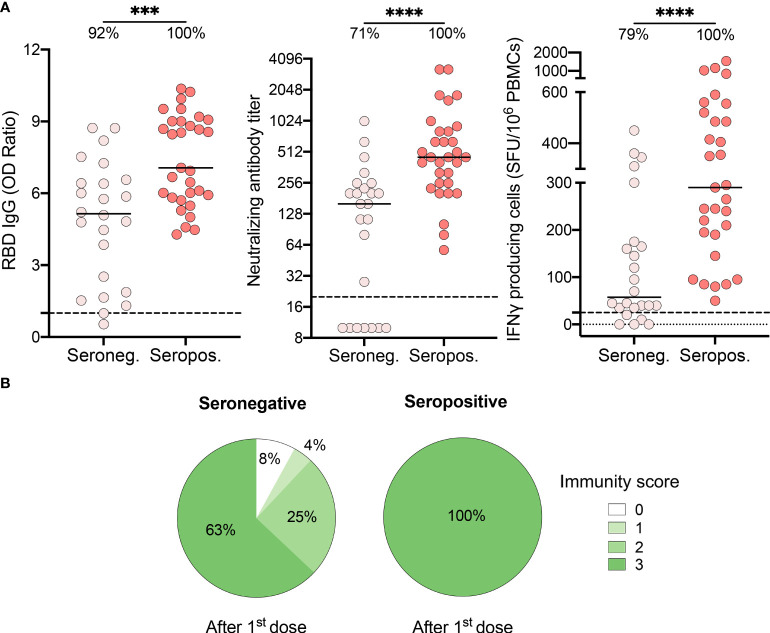
Recovered HCWs who were seronegative prior to vaccination showed suboptimal immune responses after one vaccine dose. **(A)** SARS-CoV-2 Spike RBD–specific binding IgG levels assessed by ELISA (left panel), SARS-CoV-2 neutralizing antibody titers (middle panel), IFN-γ secreting cells per million in response to SARS-CoV-2 Spike glycoprotein peptides (right panel) assessed by ELISpot for seronegative (Seroneg.) (n=24) and seropositive (Seropos.) (n=31) recovered HCWs after one dose of vaccine. Dashed black lines indicate the positive threshold value, and the percentage of HCWs with responses above positive threshold value is indicated for each condition. Horizontal bars indicate the median of each group. Statistical significance was assessed by Mann-Whitney tests. ***P <.001; ****P <.0001. **(B)** Pie charts of calculated immunity score for seronegative (left panel) and seropositive (right panel) recovered HCWs after one dose of vaccine.

In our RECOVER cohort, 24.6% (n = 140) of recovered HCWs were seronegative at enrollment in the study (23). We thus questioned if serostatus at enrollment, 3.0 ± 1.5 months prior to vaccination, could predict the immune response to the vaccine. Compared to seropositive individuals, seronegative subjects presented reduced levels of IgG (7.1 [5.8-9.0] vs. 5.2 [2.0-6.5], P=.0003), neutralizing antibodies (453.0 [254.0-806.0] vs. 160.0 [10.0-247.0], P<.0001), and IFN-γ producing cells (290.0 [190.0-520.0] vs. 57.5 [35.0-172.5], P<.0001) after one dose of vaccine (**Figure 3A**). Importantly, subjects who did not mount a positive humoral and/or cellular response after one dose of vaccine were all seronegative before vaccination. One dose of vaccine was only sufficient to boost immunity to SARS-CoV-2 in 63% of seronegative participants (**Figure 3B**). The others showed reduced capability to mount adequate neutralization of the virus and/or cell-mediated immune response, and two subjects remained nonresponsive.

### Combined effect of symptomatology and serostatus on the immunogenicity of the vaccine

**Figure 4:**
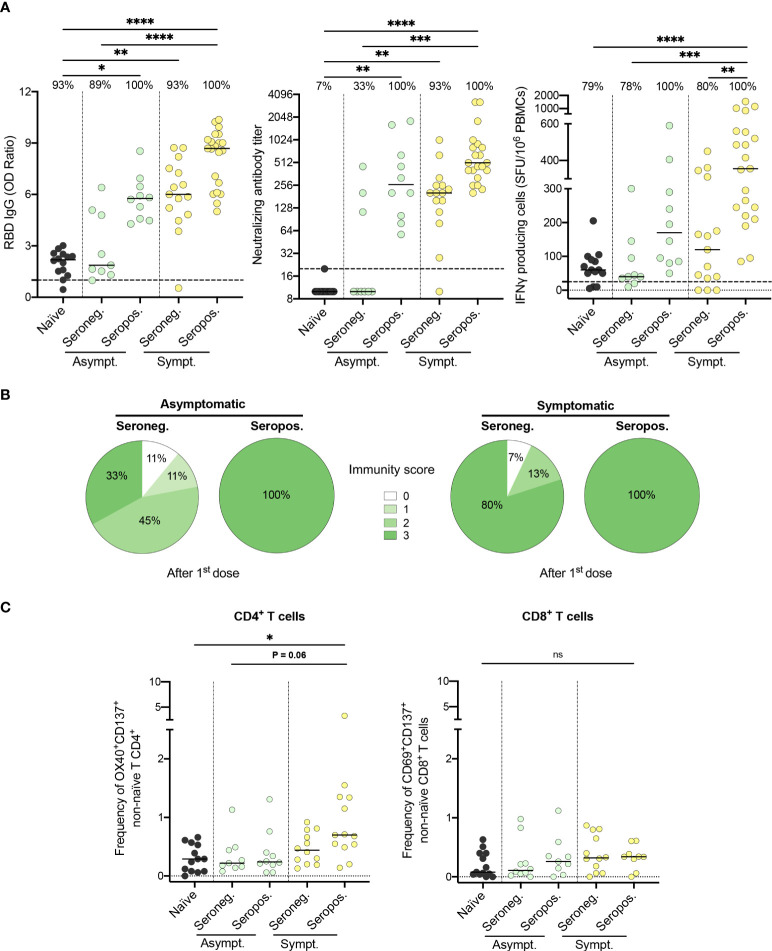
Both symptomatology during infection and serostatus prior to vaccination influence the immunogenicity of the first vaccine dose. **(A)** SARS-CoV-2 Spike RBD–specific binding IgG levels assessed by ELISA (left panel), SARS-CoV-2 neutralizing antibody titers (middle panel), and IFN-γ secreting cells per million in response to SARS-CoV-2 Spike glycoprotein peptides assessed by ELISpot (right panel) for naïve (black circles), seronegative (Seroneg.) (n=24) and seropositive (Seropos.) (n=31) recovered HCWs after one dose of vaccine. Asymptomatic recovered HCWs (Asympt.) are represented with green circles (n=19) and symptomatic HCWs (Sympt.) with yellow circles (n=36). Dashed black lines indicate the positive threshold value, and the percentage of HCWs with responses above positive threshold value is indicated for each assay. **(B)** Pie charts of calculated immunity score for asymptomatic (left panel) and symptomatic (right panel) recovered HCWs after one dose of vaccine. **(C)** Frequency of SARS-CoV-2−Spike-specific T cells measured as percentage of OX40^+^CD137^+^ non-naïve CD4^+^ (left panel) and CD69^+^CD137^+^ non-naïve CD8^+^ T cells (right panel) after stimulation of PBMCs with CD4-S mega pool of peptides from the Spike glycoprotein for naïve (n=13), asymptomatic seronegative (n=9) or seropositive (n=10) and symptomatic seronegative (n=12) or seropositive (n=13) recovered HCWs after one dose of vaccine. Horizontal bars indicate the median of each group. Kruskal-Wallis tests assessed statistical significance **(A-C)**. Not significant (ns) P >.05; *P < .05; **P <.01; ***P < .001; ****P < .0001.

Most previously infected HCWs who did not develop an immunity score of three after the first dose of vaccine had an asymptomatic SARS-CoV-2 infection (6 out of 9; **Figure 2B**). To better depict the impact of seropositivity and symptomatology on the immune response to one dose of vaccine, we compared levels of induced immunity within each subgroup. Symptomatic individuals who remained seropositive following natural infection developed the most vigorous humoral and cellular immune response to one dose of vaccine (**Figure 4A, B**). Furthermore, their frequency of SARS-CoV-2 specific CD4^+^ T cells, as assessed by the expression of activation markers, was the highest (0.7 [0.5-1.3] vs. 0.3 [0.1-0.6], P=.02) for naïve subjects (**Figure 4C**). In contrast, asymptomatic individuals who were seronegative at enrollment had significantly lower anti-RBD IgG levels (1.9 [1.4-4.9] vs. 8.7 [6.4-9.4], P<.0001), neutralizing antibody titers (10.0 [10.0-157.5] vs. 508.0 [361.5-855.5], P<.001) as well as IFN-γ secreting cells (40.0 [27.5-120.0] vs. 355.0 [232.5-557.5] SFU/10^6^ PBMCs, P<.001) following one dose of the vaccine, compared to seropositive symptomatic subjects. Notably, this subgroup of asymptomatic seronegative individuals was the only group to demonstrate an immune response following one dose of vaccine that was similar to naïve individuals. In fact, prior to vaccination, these individuals lacked immunological markers of previous antigen encounter, as did SARS-CoV-2 naïve subjects (**Figure S7 in Supplemental 1**).

### A two-dose primary series of mRNA vaccine is required in previously infected individuals who did not experience symptoms

**Figure 5:**
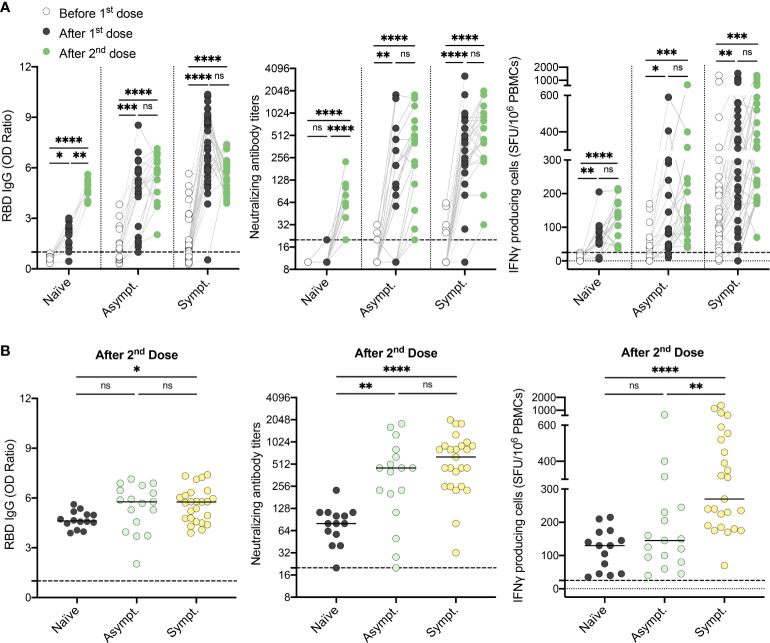
Two doses of vaccine are required for individuals who recovered from an asymptomatic SARS-CoV-2 infection to generate a global and coordinated adaptive immune response. **(A)** SARS-CoV-2 Spike RBD–specific binding IgG levels assessed by ELISA (left panel), SARS-CoV-2 neutralizing antibody titers (middle panel), IFN-γ secreting cells per million in response to SARS-CoV-2 Spike glycoprotein peptides (right panel) assessed by ELISpot for naïve (n=14), asymptomatic (n=17) and symptomatic recovered HCWs (n=25) before (white circles), after one dose (black circles) and after two doses of vaccine (green circles). **(B)** SARS-CoV-2 Spike RBD–specific binding IgG levels assessed by ELISA (left panel), SARS-CoV-2 neutralizing antibody titers (middle panel), and IFN-γ secreting cells per million in response to SARS-CoV-2 Spike glycoprotein peptides assessed by ELISpot (right panel) for naïve (black circles), asymptomatic (n=17) and symptomatic (n=25) recovered HCWs after two doses of vaccine. Dashed black lines indicate the positive threshold value. Horizontal bars indicate the median of each group. Kruskal-Wallis tests assessed statistical significance. Not significant (ns) P >.05; *P <.05; **P <.01; ***P <.001; ****P <.0001.

Among HCWs who recovered from SARS-CoV-2 infection, 16% did not demonstrate a well-coordinated humoral and cellular immune response after one vaccine dose (**Figure 1B**), with asymptomatic, seronegative subjects being the least responsive. Therefore, we assessed humoral and cellular responses after the second dose of vaccine. Strikingly, all subjects displayed a strong humoral and cellular immune response after two doses of vaccine, including naïve and asymptomatic participants, as demonstrated by elevated anti-RBD IgG levels, neutralizing capability, as well as IFN-γ secreting cells (**Figure 5A** and **Figure S8 in Supplemental 1**). After two doses of vaccine, naïve individuals still showed slightly lower anti-RBD IgG levels compared to both asymptomatic and symptomatic recovered individuals (**Figure 5B**). Cellular responses remained lower in asymptomatic HCWs compared to their symptomatic counterparts (**Figure 5B**). Our results collectively demonstrate that the severity of initial infection leads to the development of a more robust and persistent immune memory response after a vaccination challenge. In contrast, individuals who did not experience symptoms during infection with SARS-CoV-2 are less likely to develop long-term humoral and cellular immunity.

## Discussion

The ultimate goal of vaccination is to generate broad and long-lasting immune responses that will protect the host from severe clinical outcomes if infected. The most appropriate vaccination regimen to be used in previously infected individuals remains undefined, leading to discrepancies in public health recommendations worldwide. Based on prior studies suggesting that previously infected individuals did not benefit from their second dose of mRNA vaccine (18, 22), many countries have moved forward with the recommendation that a single dose of mRNA vaccine was sufficient for individuals who were previously infected (9). In this report, we demonstrate that one dose of vaccine is sufficient to induce sustained humoral and cellular immune responses in 92% of previously infected individuals who experienced symptoms during their infection. In contrast, only 69% in individuals who remained asymptomatic during their primary infection mounted robust immune responses to one dose of vaccine. In fact, infected individuals who remained asymptomatic and were seronegative at enrollment in our study harbored an immune response comparable to naïve subjects before and after vaccination.

Our results differ from one American HCWs cohort that concluded that humoral immune responses shortly after one mRNA vaccine dose were comparable in individuals who recovered from symptomatic and asymptomatic infections (34). However, HCWs from that cohort were all seropositive at the time of vaccination. Here, we show that previously infected but asymptomatic individuals who harbor a negative serostatus at vaccination mount reduced serum neutralization capabilities and cell-mediated memory responses following one vaccine dose. Furthermore, our study is the first one to describe both cellular and humoral immunity developed after vaccination in recovered subjects from an asymptomatic infection.

Knowledge gained from this study is particularly important in the current context of booster vaccination and protection against recent variants of concerns (VOCs). Waning immunity has been reported six months after a two-dose primary series in naïve individuals (35, 36). In these naïve individuals, three doses of vaccine increase protection against symptomatic and severe B.1.617.2 (Delta) and B.1.1.529 (Omicron) infections (37–42). Notably, despite strengthened immune responses following the booster dose, the capacity to neutralize the B.1.1.529 variant appears weaker (43–46). A German study reported that neutralizing antibodies against the Omicron variant were highest in individuals who had encountered the antigen three times, whether it was from receiving three doses of mRNA vaccine for uninfected individuals or two doses for individuals who had been infected with SARS-CoV-2 either prior to or subsequent to vaccination (45). Based on our current report, we want to highlight that this affirmation is true only in subjects who experienced symptoms during infection. Indeed, our results suggest that infected individuals who did not develop symptoms should receive additional booster doses, as should previously uninfected individuals, since we demonstrated that a fraction of these individuals harbors immune responses to vaccination comparable to naïve individuals. Further studies will be required to evaluate the vaccine requirements for boosters in recently infected Omicron cases, as people infected with this variant are more likely to be asymptomatic (47).

Limitations of this study are mainly related to the fact that results were obtained from a cohort of primarily middle-aged Caucasian females infected with SARS-CoV-2 eight months before vaccination. The immunization strategy in our province led to an important delay of sixteen weeks between the first and the second doses in these previously infected individuals, compared to ten weeks in uninfected individuals, which should be taken into consideration when interpreting the results. Indeed, an extended vaccination regimen was shown to favor better immune memory (48). It therefore remains to be evaluated if a shorter interval between infection and vaccination, or between the two vaccine doses, lead to similar results. In addition, samples available before and after each dose of vaccine being an important constraint, the time window between vaccination and analysis had to be extended for previously infected HCWs, while uninfected individuals were closer to one-month post-vaccination. Further, the protection induced by booster doses against infection by VOCs in previously infected, but asymptomatic individuals remain to be evaluated. The low frequency of asymptomatic individuals in our HCWs cohort and attrition with time prevented us to evaluate persistence and expansion of immune memory before and after booster vaccination. This study did not assess the impact of other approved vaccines against SARS-CoV-2 (49, 50), as primary series or boosters, as well as natural infection with emerging VOCs, as most of the cohort was infected at the beginning of 2020, with the original strain (51). While the immunity score we developed allows to easily report on the multi-functionality of the immune response induced by hybrid immunity, it does not report on the strength of the response. Evidence is currently lacking to establish the threshold at which humoral and cellular responses are optimal for heightened protection. This limitation could eventually be overcome by refining our metric to be more informative on protective immunity based on new evidence and evaluation of larger cohorts. Detailed functional, transcriptomic and repertoire analyses will be essential to fully grasp the immune memory differences between individuals who experienced symptoms or not during infection.

## Conclusions

This report reveals the critical importance of the impact of an individual’s symptomatology at the time of infection and serostatus at the time of vaccination on the capacity to mount adequate immune protection, including cell-mediated responses. In contrast to individuals who were symptomatic during infection, we demonstrated that a third of the individuals who recovered from an asymptomatic infection responded like naïve individuals. We recommend that the two-dose primary series, and subsequent boosters, be offered to all previously infected individuals who were asymptomatic, in order to elicit a global and sustained adaptive immune response following mRNA vaccination.

## Data availability statement

The raw data supporting the conclusions of this article will be made available by the authors, without undue reservation. Inquiries can be directed to the corresponding author.

## Ethics statement

The studies involving human participants were reviewed and approved by Research Ethics Board (REB) at the Sainte-Justine University Hospital and Research Center. The patients/participants provided their written informed consent to participate in this study.

## Author contributions

HD, CQ: Conceptualized the project, designed the study and experiments, and obtained funding. SN, BB, JuC, HR, M-EH: Performed the experiments. SN, BB, HD, MB: Analyzed the data. SN, BB, HD: Interpreted the results and prepared the figures. KA, DM: Performed the clinical follow-up of the study. SN, BB, HD: Wrote the manuscript. CQ, VG, MC, PS, GS, JaC, YL, MB, GB: Edited the manuscript and provided critical input. All authors contributed to the article and approved the submitted version.

## Funding

This work was funded by the Canadian Institutes of Health Research (CIHR) (VR2172712) and the Public Health Agency of Canada, through the Vaccine Surveillance Reference Group and the COVID-19 Immunity Task Force. This work was also supported by NIH contract 75N93019C00065 (A.S, D.W).

## Acknowledgments

We want to thank Valérie Villeneuve, Jocelyne Ayotte, and the Rare Pediatric Disease (RaPID) Biobank personnel at the Sainte-Justine University Hospital and Research Center for their technical help and expertise. Special thanks to Fazia Tadount and the team at the Vaccine Study Center for coordinating the RECOVER studies, and to Zineb Laghdir for administrative support for the RECOVER studies. We are grateful to the nurses and research coordinators at each University Hospital for their involvement in this study and the healthcare workers who agreed to participate in this research. We also thank Alessandro Sette and Daniela Weiskopf for providing the mega pool of peptides utilized in the AIM assays.

## Conflict of interest

The authors declare that the research was conducted in the absence of any commercial or financial relationships that could be construed as a potential conflict of interest.

## Publisher’s note

All claims expressed in this article are solely those of the authors and do not necessarily represent those of their affiliated organizations, or those of the publisher, the editors and the reviewers. Any product that may be evaluated in this article, or claim that may be made by its manufacturer, is not guaranteed or endorsed by the publisher.
