# Supplementary material for: Symptomatology during previous SARS-CoV-2 infection and serostatus before vaccination influence the immunogenicity of BNT162b2 COVID-19 mRNA vaccine
Source: Front Immunol. 2022 Oct 14;13:930252. doi: 10.3389/fimmu.2022.930252 (PMC9614167; doi:10.3389/fimmu.2022.930252)
Supplement: Supplementary file 1 [file DataSheet_1.pdf]

**Supplementary Information for :**

# Symptomatology during previous SARS-CoV-2 infection and serostatus before vaccination influence the immunogenicity of BNT162b2 COVID-19 mRNA vaccine


## AUTHORS

Sabryna Nantel[1,2] (Ph.D. candidate), Benoîte Bourdin[1] (Ph.D.), Kelsey Adams[3] (M.Sc.), Julie Carbonneau[4,5] (M.Sc.), Henintsoa Rabezanahary[4,5,12] (Ph.D.), Marie-Ève Hamelin[4,5] (Ph.D.), Deirdre McCormack[3] (B.Sc.(N)), Patrice Savard[2,6] (M.D.), Yves Longtin[7] (M.D.), Matthew P. Cheng[8] (M.D.), Gaston De Serres[5,9,10] (M.D.), Jacques Corbeil[5,11] (Ph.D.), Vladimir Gilca[5,9,10] (M.D.), Mariana Baz[4,5,12] (Ph.D.), Guy Boivin[4,5] (M.D.), Caroline Quach[2,3,*] (M.D.), Hélène Decaluwe[1,2,13,*] (M.D.)

## AFFILIATIONS

[1] Cytokines and Adaptive Immunity Lab, Sainte-Justine University Hospital and Research Center, Montréal, QC, Canada
[2] Microbiology, Infectiology and Immunology Department, Faculty of Medicine, University of Montréal, QC, Canada
[3] Clinical Department of Laboratory Medicine, Infection Prevention and Control, Sainte-Justine University Hospital and Research Center, Montréal, QC, Canada
[4] Infectious Disease Research Center, Université Laval, Québec City, QC, Canada
[5] Centre Hospitalier Universitaire de Québec - Université Laval Research Center, Québec City, QC, Canada
[6] Immunopathology Department, Montreal University Hospital and Research Center, Montréal, QC, Canada
[7] Infectious Diseases Service, Department of Medicine, Jewish General Hospital, Montréal, QC, Canada
[8] Divisions of Infectious Diseases and Medical Microbiology, Departments of Medicine and Laboratory Medicine, McGill University Health Center, Montréal, QC, Canada
[9] Biological and Occupational Risk, Institut National de Santé Publique du Québec, Québec City, QC, Canada
[10] Preventive and Social Medicine Department, Université Laval, Québec City, QC, Canada
[11] Molecular Medicine Department, Université Laval, Québec City, QC, Canada
[12] Microbiology, Infectiology and Immunology Department, Université Laval, Québec City, QC, Canada
[13] Pediatric Immunology and Rheumatology Division, Department of Pediatrics, University of Montréal, QC, Canada

* Designates (2) shared senior authorship

## CORRESPONDING AUTHOR

Hélène Decaluwe, M.D. Ph.D.
Sainte-Justine University Hospital and Research Center
3175, Chemin de la Côte-Sainte-Catherine, Montréal, QC, Canada (H3T 1C5)
1-514-345-4931
helene.decaluwe@umontreal.ca

## SUPPLEMENTAL FIGURES AND FIGURE LEGENDS

3 Supplemental Tables

**Supplemental Table 1.** Characteristics of naïve and recovered HCWs included in assays.

**Supplemental Table 2.** Intervals for assessment of naïve individuals and HCWs who recovered from previous asymptomatic or symptomatic SARS-CoV-2 infection and were vaccinated with BNT162b2 mRNA vaccine.

**Supplemental Table 3.** Antibodies used for flow cytometry analysis.

8 Supplemental Figures

**Figure S1.** Timeline of blood collection for humoral and cellular analyses of vaccinated HCWs who recovered from SARS-CoV-2 infection or individuals naïve to SARS-CoV-2 before vaccination.

**Figure S2.** Gating strategy for AIM assays and representative dot plots.

**Figure S3.** The strength of the humoral response in recovered HCWs from SARS-CoV-2 infection is correlated with symptomatology during infection.

**Figure S4.** Recovered HCWs from SARS-CoV-2 infection maintain strong and stable SARS-CoV-2-specific cellular memory up to 11 months after infection.

**Figure S5.** Recovered HCWs develop much stronger SARS-CoV-2-specific humoral and cellular immune responses than naïve individuals after one dose of vaccine.

**Figure S6.** Humoral and cellular responses that developed after one dose of vaccine are correlated in HCWs who recovered from SARS-CoV-2 infection.

**Figure S7.** Previously infected but seronegative individuals show limited humoral and cellular response before vaccination.

**Figure S8.** Previously infected individuals develop stronger humoral and cellular responses to mRNA vaccination than naïve subjects.

## SUPPLEMENTAL TABLES

**Supplemental Table 1. Characteristics of naïve and recovered HCWs included in assays.**

| | Number (%) of naïve individuals (n=14) | Number (%) of recovered HCWs (n=55) |
|---|---|---|
| **Demographics** | | |
| **Sex** | | |
| Male | 5 (36) | 20 (36) |
| Female | 9 (64) | 35 (64) |
| **Age (y)** | | |
| Mean ± SD | 43 ± 9 | 41.8 ± 12.3 |
| Median [Min, Max] | 41 [27, 57] | 44.6 [19.4, 67.1] |
| **Ethnicity** | | |
| Caucasian | 13 (93) | 45 (82) |
| Middle Eastern | 0 (0) | 1 (2) |
| Latin American | 0 (0) | 2 (4) |
| Asian | 1 (7) | 4 (7) |
| Black or African American | 0 (0) | 3 (5) |
| **Symptomatology at initial infection** | | |
| Asymptomatic / WHO Score of 1 | n/a | 19 (35) |
| Symptomatic / WHO Score of 2-3 | n/a | 30 (54) |
| Symptomatic / WHO Score of 4-5 | n/a | 6 (11) |
| **Time since initial infection at enrollment (m)** | | |
| Mean ± SD | n/a | 5.3 ± 1.9 |
| Median [Min, Max] | n/a | 5.3 [0.7, 9.3] |
| **Serology Status at Enrollment** | | |
| **Seronegative** | n/a | 24 (44) |
| **Seropositive** | n/a | 31 (56) |

Abbreviations : SD, Standard Deviation; y, years; m, months.

**Supplemental Table 2. Intervals for assessment of naïve individuals and HCWs who recovered from previous asymptomatic or symptomatic SARS-CoV-2 infection and were vaccinated with BNT162b2 mRNA vaccine.**

| | Number (%) of naïve individuals (n=14) | Number (%) of asymptomatic HCWs (n=19) | Number (%) of symptomatic HCWs (n=36) |
|---|---|---|---|
| **Vaccination received** | | | |
| Pfizer BioNTech | 14 (100) | 9 (100) | 15 (100) |
| **Time since initial infection at first analysis (prior to vaccination; m)** | | | |
| Mean ± SD | n/a | 6.8 ± 2.3 | 7.1 ± 2.2 |
| Median [Min, Max] | n/a | 7.1 [0.7, 10.5] | 7.3 [3.2, 13.1] |
| **Time since initial infection at first vaccine dose (m)** | | | |
| Mean ± SD | n/a | 8.4 ± 2.4 | 8.4 ± 2.3 |
| Median [Min, Max] | n/a | 8.7 [0.8, 12.5] | 8.5 [3.3, 13.1] |
| **Time since first dose at analysis (d)** | | | |
| Mean ± SD | 29.1 ± 2.1 | 47.4 ± 29.2 | 43.8 ± 23.0 |
| Median [Min, Max] | 28 [27, 33] | 41 [10, 93] | 34.5 [10, 91] |
| **Time since initial infection at second vaccine dose (m)** | | | |
| Mean ± SD | n/a | 12.4 ± 1.8 | 12.4 ± 2.9 |
| Median [Min, Max] | n/a | 12.5 [7.3, 15] | 12.7 [5, 16.4] |
| **Time since second dose at analysis (d)** | | | |
| Mean ± SD | 35.2 ± 4.9 | 51.3 ± 31.8 | 60.6 ± 29.4 |
| Median [Min, Max] | 34 [26.45] | 44 [13, 121] | 58 [23, 116] |
| **Time between first and second vaccine dose (w)**[#] | | | |
| Mean ± SD | 9.5 ± 0.8 | 15.3 ± 3.1 | 16.8 ± 6.7 |
| Median [Min, Max] | 9.6 [8.0, 11.3] | 15.7 [8.6, 23] | 16.1 [3, 32.3] |

[#]: $p < 0.0001$

Abbreviations : SD, Standard Deviation; w, weeks; m, months; d, days.

**Supplemental Table 3. Antibodies used for flow cytometry analysis.**

| Antibodies | Company | Clone | Cat# |
|---|---|---|---|
| BUV395 Mouse Anti-Human CD3 | BD | SK7 | 564001 |
| V500 Mouse Anti-Human CD4 | BD | RPA-T4 | 560768 |
| AF700 Mouse Anti-Human CD8 | BD | RPA-T8 | 557945 |
| BUV737 Mouse Anti-Human CCR7 | BD | 2-L1-A | 749676 |
| eF450 Mouse Anti-Human CD45-RA | Invitrogen | HI100 | 48-0458-42 |
| APC Mouse Anti-Human CD137 | BioLegend | 4B4-1 | 309810 |
| PE-Cy7 Mouse Anti-Human OX40 | BioLegend | Ber-ACT35 | 350012 |
| BV785 Mouse Anti-Human CD25 | BioLegend | BC96 | 302638 |
| PE Mouse Anti-Human CD69 | BD | FN50 | 555531 |

**Figure S1. Timeline of blood collection for humoral and cellular analyses of vaccinated HCWs who recovered from SARS-CoV-2 infection or individuals naïve to SARS-CoV-2 before vaccination.**

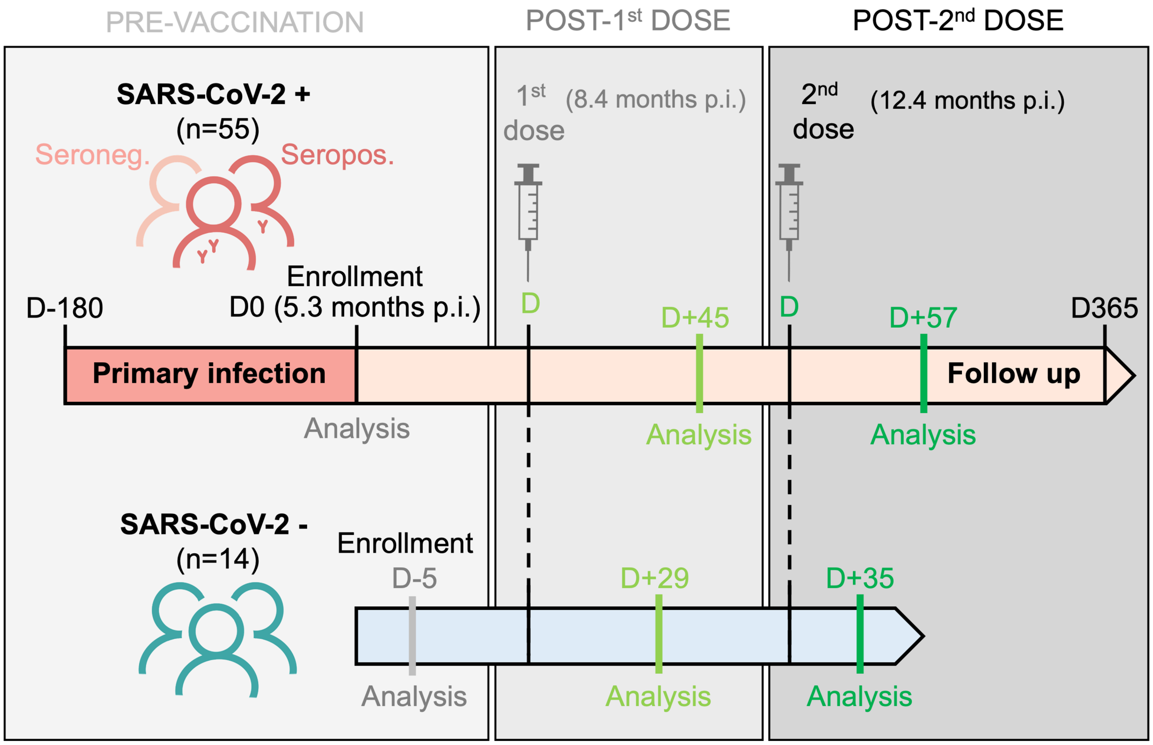


Blood samples for humoral and cell-mediated immunity were collected at enrollment (D0), around D+45 after the first dose and around D+57 after the second dose of vaccine for seronegative (Seroneg.) and seropositive (Seropos.) HCWs recovered from SARS-CoV-2 infection (SARS-CoV-2 +, n=55). For 14 naïve participants (SARS-CoV-2 -), blood samples were collected before their first dose of vaccine (D-5/D0), around D+29 after the first dose and around D+35 after the second dose.

**Figure S2. Gating strategy for AIM assays and representative dot plots.**

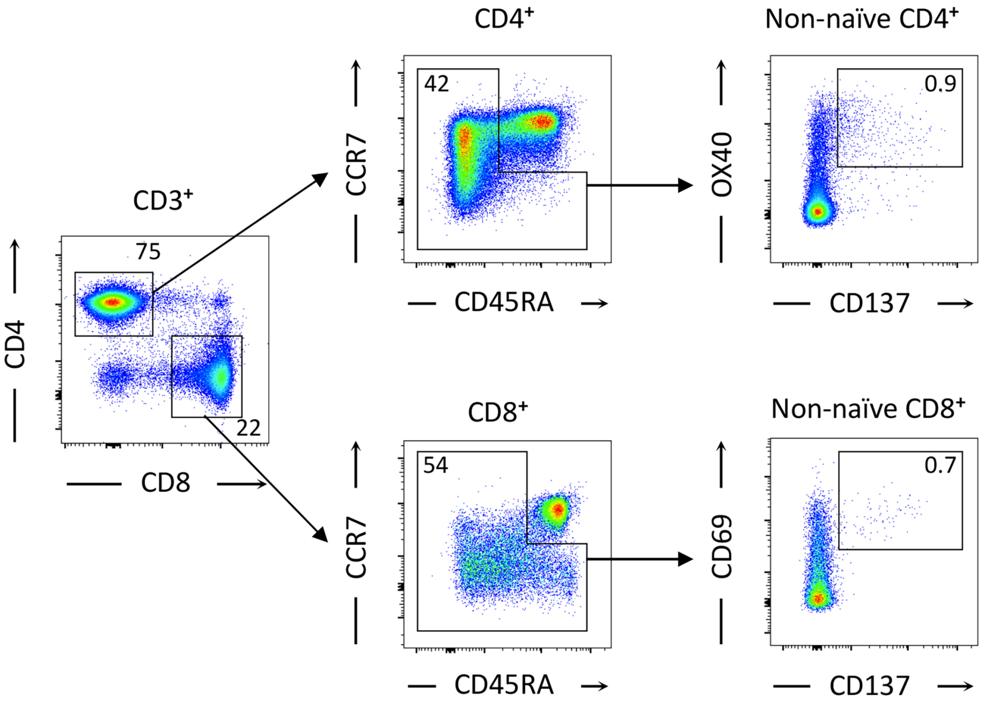


Gating used to identify SARS-CoV-2−specific $CD4^+$ ($OX40^+CD137^+$) and $CD8^+$ ($CD69^+CD137^+$) T cells in recovered HCWs from SARS-CoV-2 infection or naïve to the infection vaccinees after stimulation of PBMCs with CD4-S mega pool of peptides from the Spike glycoprotein. The percentage of events is indicated within $AIM^+$ (double-positive) quadrant.

**Figure S3. The strength of the humoral response in recovered HCWs from SARS-CoV-2 infection is correlated with symptomatology during infection.**

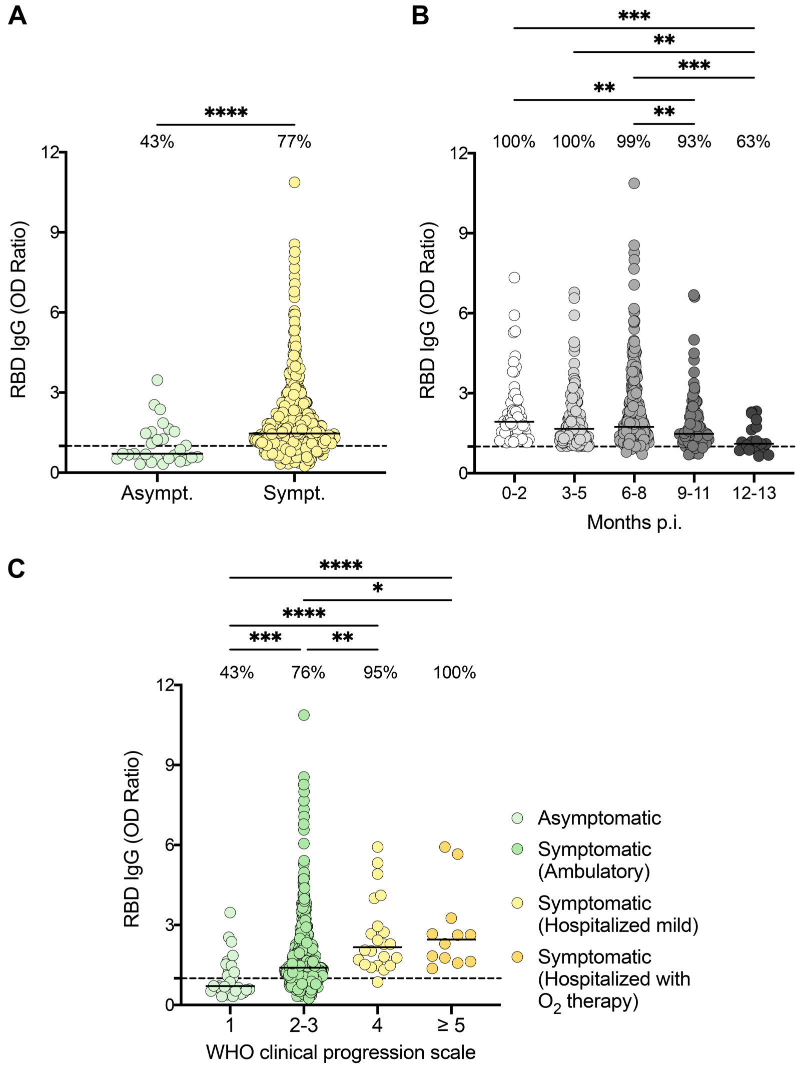


(**A**) SARS-CoV-2 Spike RBD–specific binding IgG levels assessed by ELISA for 569 recovered HCWs classified based on their symptomatology upon infection. (**B**) Kinetic of RBD-binding IgG levels over one-year post infection in participants who were seropositive at enrollment. (**C**) SARS-CoV-2 Spike RBD–specific binding IgG levels assessed by ELISA for 569 recovered HCWs classified according to WHO clinical progression scale. (**A**) Asymptomatic (Asympt.): n=28, Symptomatic (Sympt.): n=541. (**B**) 0-2 months: n=57, 3-5: n=175, 6-8: n=297, 9-11: n=108, 12-13: n=19. (**C**) WHO clinical progression scale 1: n=28, 2-3: n=507, 4: n=22, ≥5: n=12. The black dashed lines indicate the positive threshold value, and the percentage of seropositive participants is indicated for each subgroup. Horizontal bars indicate the median of each group. Statistical significance was assessed by Mann-Whitney (**A**) and Kruskal-Wallis (**B-C**) tests. *P <.05; **P <.01; ***P <.001; ****P <.0001.

**Figure S4. Recovered HCWs from SARS-CoV-2 infection maintain strong and stable SARS-CoV-2-specific cellular memory up to 11 months after infection.**

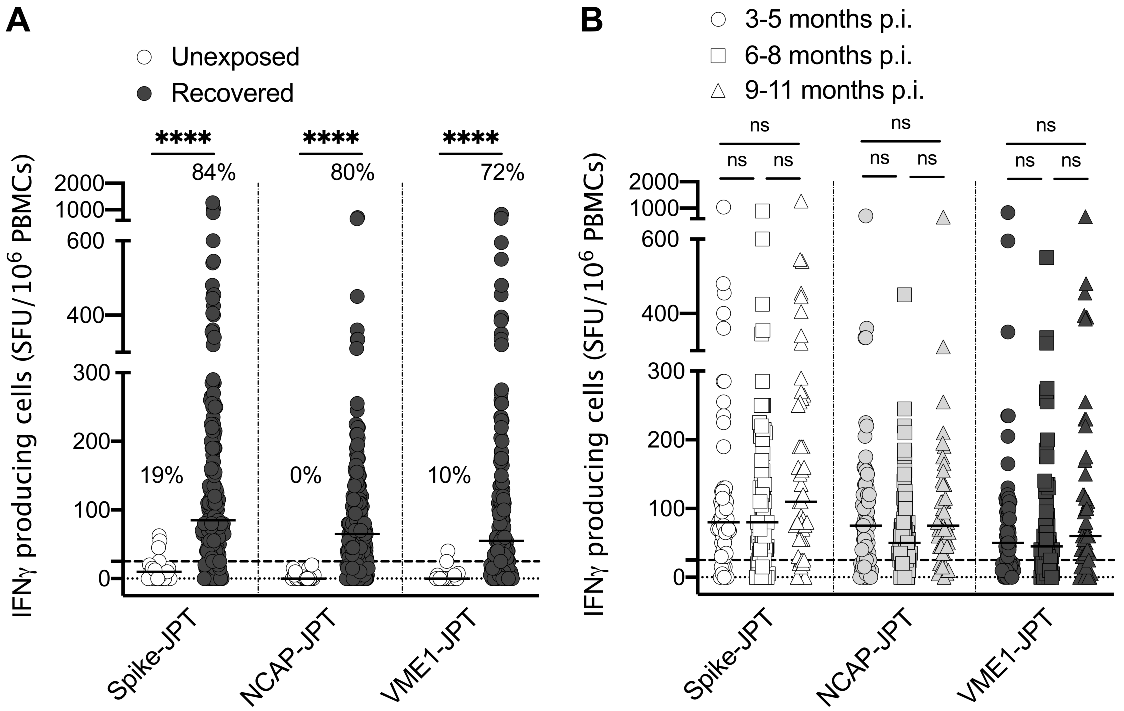


(**A-B**) IFN-γ producing cells per million in response to SARS-CoV-2 Spike glycoprotein, nucleocapsid protein (NCAP) and membrane protein (VME1) mega pools of peptides assessed by ELISpot for naïve (white circles) (n=21) and recovered individuals (black circles) (n=200) at enrollment (**A**) and kinetic of the response one-year post infection (3-5 months p.i.: n=63, 6-8 months p.i.: n=70, 9-11 months p.i.: n=48) (**B**). Black dashed lines indicate the positive threshold value, and the percentage of individuals with responses above positive threshold value is indicated for each subgroup and time point. Horizontal bars indicate the median of each group. Statistical significance was assessed by Mann-Whitney (**A**) and Kruskal-Wallis (**B**) tests. Not significant (ns) P >.05; ****P <.0001.

**Figure S5. Recovered HCWs develop much stronger SARS-CoV-2-specific humoral and cellular immune responses than naïve individuals after one dose of vaccine.**

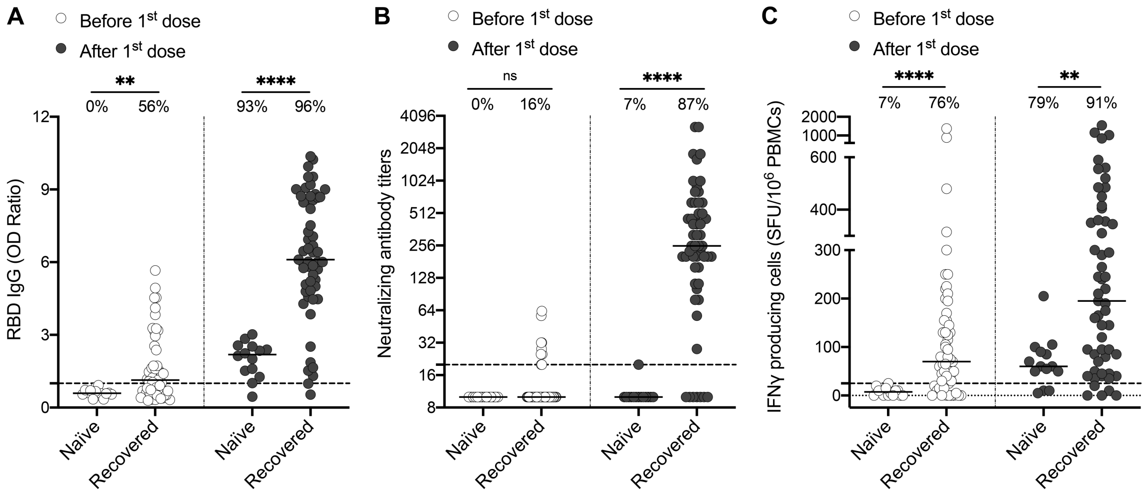


(**A-C**) SARS-CoV-2 Spike RBD–specific binding IgG levels assessed by ELISA (**A**), SARS-CoV-2 neutralizing antibody titers (**B**), IFN-γ producing cells per million in response to SARS-CoV-2 spike glycoprotein peptides stimulation (**C**) assessed by ELISpot for naïve (n=14) and recovered HCWs (n=55) before (white circles) and after one dose of vaccine (black circles). Black dashed lines indicate the positive threshold value, and the percentage of individuals with responses above positive threshold value is indicated for each subgroup and time point. Horizontal bars indicate the median of each group. Statistical significance was assessed by Mann-Whitney tests. Not significant (ns) P >.05; **P <.01; ****P <.0001.

**Figure S6. Humoral and cellular responses developed after one vaccine dose are correlated in HCWs who recovered from SARS-CoV-2 infection.**

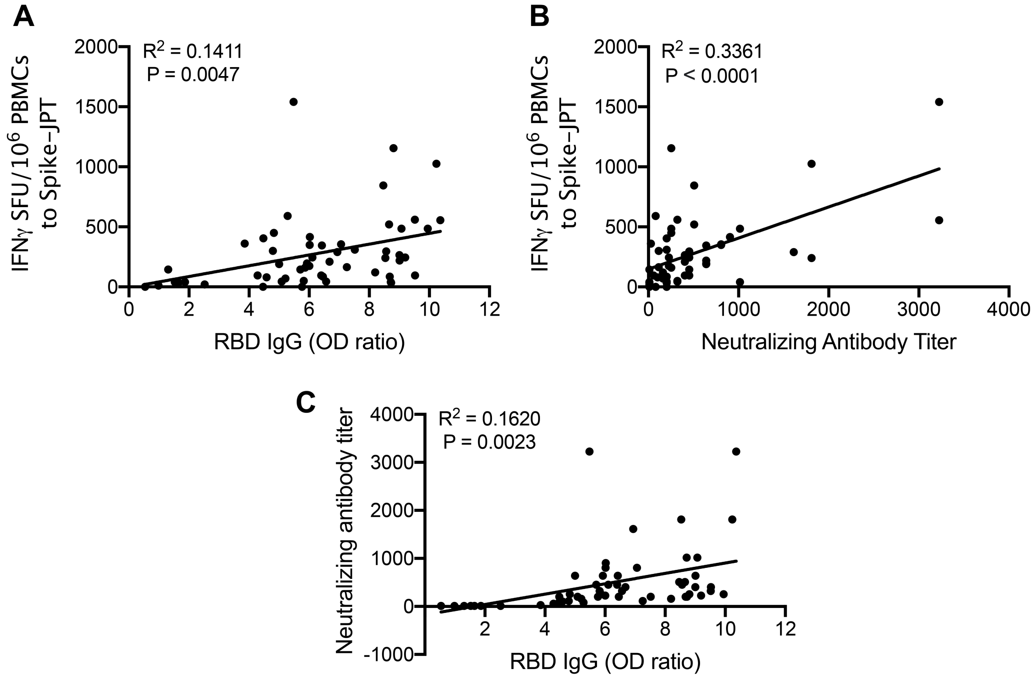


(**A-C**) Correlation between IFN-γ secreting cells per million in response to SARS-CoV-2 Spike glycoprotein peptides and SARS-CoV-2 Spike RBD–specific binding IgG levels (**A**), IFN-γ secreting cells per million in response to SARS-CoV-2 Spike glycoprotein peptides and SARS-CoV-2 neutralizing antibody titers (**B**), SARS-CoV-2 neutralizing antibody titers and SARS-CoV-2 Spike RBD–specific binding IgG levels (**C**) in recovered HCWs from SARS-CoV-2 infection after one dose of vaccine (n=67). Correlation was assessed by Pearson correlation coefficient. Coefficient $R^2$ and P-values are shown.

**Figure S7. Previously infected but seronegative individuals show limited humoral and cellular response before vaccination.**

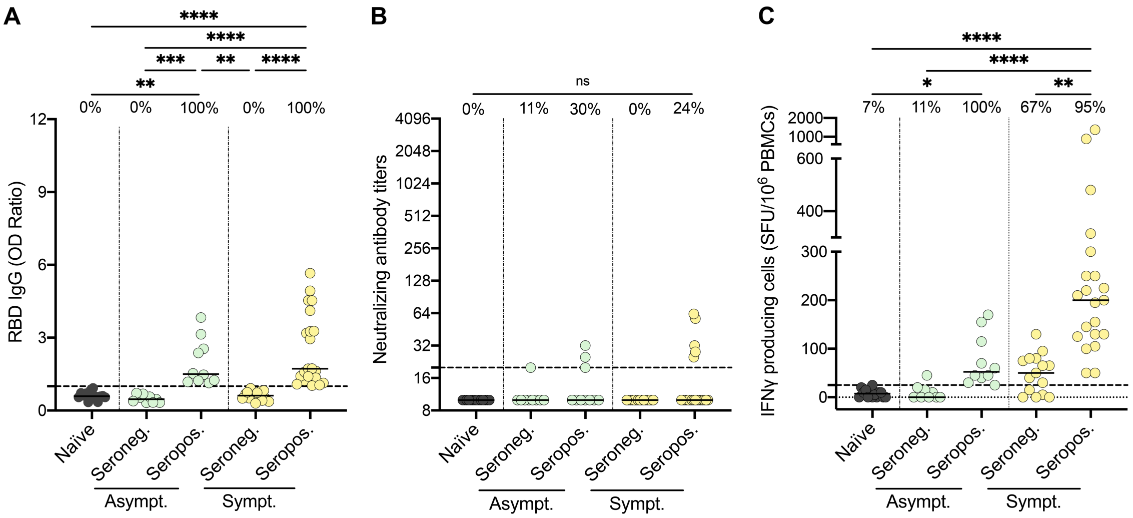


(**A-C**) SARS-CoV-2 Spike RBD–specific binding IgG levels assessed by ELISA (**A**), SARS-CoV-2 neutralizing antibody titers (**B**), IFN-γ secreting cells per million in response to SARS-CoV-2 Spike glycoprotein peptides stimulation (**C**) assessed by ELISpot for seronegative (Seroneg.) (n=18) and seropositive (Seropos.) (n=37) recovered HCWs after natural infection. Asymptomatic recovered HCWs (Asympt.) are represented with green circles (n=17) and symptomatic HCWs (Sympt.) with yellow circles (n=38). Naïve individuals are shown as negative control (black circles). Black dashed lines indicate the positive threshold value, and the percentage of HCWs with responses above positive threshold value is indicated for each subgroup. Horizontal bars indicate the median of each group. Statistical significance was assessed by Kruskal-Wallis tests. Not significant (ns) P >.05; *P <.05; **P <.01; ***P <.001; ****P <.0001.

**Figure S8. Previously infected individuals develop stronger humoral and cellular responses to mRNA vaccination than naïve subjects.**

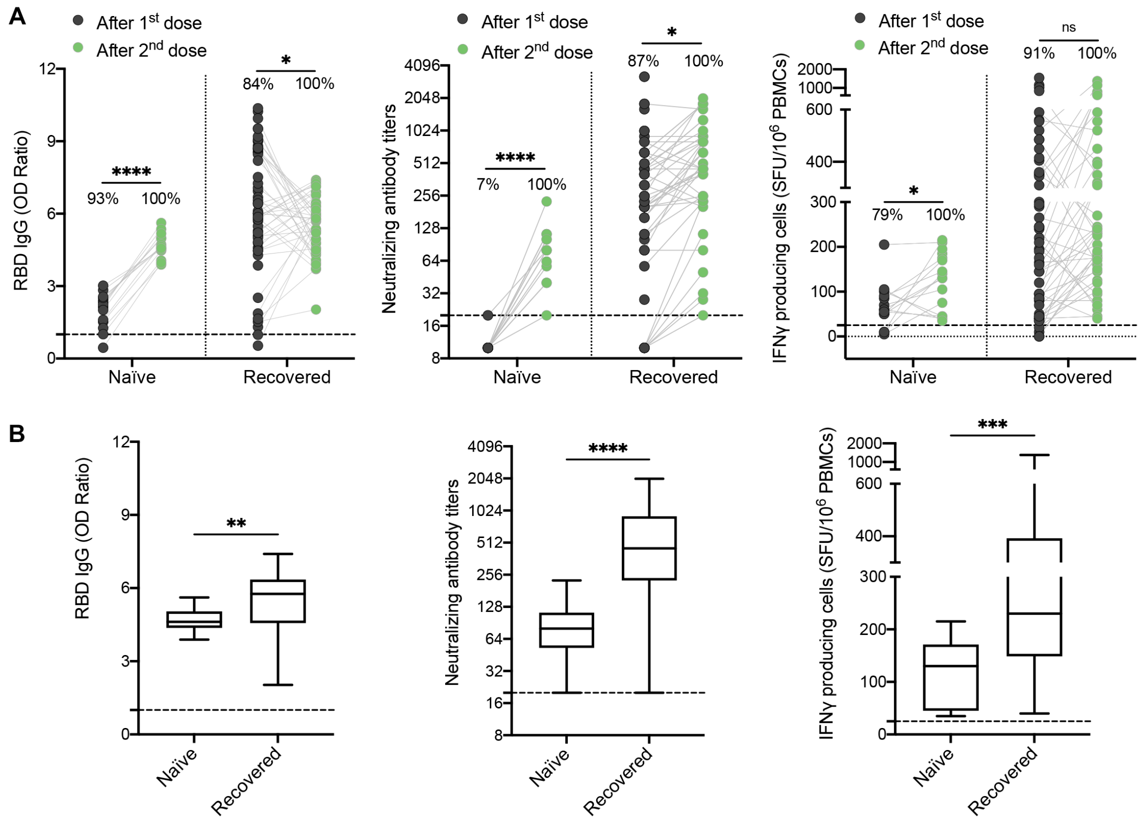


(**A**) SARS-CoV-2 Spike RBD–specific binding IgG levels assessed by ELISA (left panel), SARS-CoV-2 neutralizing antibody titers (middle panel), IFN-γ secreting cells per million in response to SARS-CoV-2 Spike glycoprotein peptides stimulation (right panel) assessed by ELISpot for naïve (n=14) and recovered individuals (n=55) after one dose (black circles) and two doses of vaccine (green circles). (**B**) SARS-CoV-2 Spike RBD–specific binding IgG levels assessed by ELISA (left panel), SARS-CoV-2 neutralizing antibody titers (middle panel), IFN-γ secreting cells per million in response to SARS-CoV-2 Spike glycoprotein peptides stimulation (right panel) assessed by ELISpot for naïve (n=14) and recovered individuals (n=42) after two doses of vaccine. Black dashed lines indicate the positive threshold value, and the percentage of individuals with responses above positive threshold value is indicated for each time point. (**B**) Boxes delimitate the IQR 25th – 75th percentiles and the median are identified by the horizontal line in each box. Error bars indicate the minimal and maximal values. Statistical significance was assessed by Mann-Whitney tests. Not significant (ns) P >.05; *P <.05; **P <.01; ***P <.001; ****P <.0001.
